# Antifibrogenic and apoptotic effects of Ocoxin in cultured rat hepatic stellate cells

**DOI:** 10.1007/s13105-022-00878-5

**Published:** 2022-03-03

**Authors:** Marina Ruiz de Galarreta, Elena Arriazu, María P. Pérez de Obanos, Eduardo Ansorena, María J. Iraburu

**Affiliations:** Department of Biochemistry and Genetics, University of Navarra, 31008 Pamplona, Spain

**Keywords:** Ocoxin, Apoptosis, Hepatic stellate cells, Liver fibrosis, p38 MAPK

## Abstract

Ocoxin is a nutritional supplement that has been shown to exert antioxidant and immunomodulatory responses in patients with chronic hepatitis C. The present work aimed to determine the effects of Ocoxin on activated hepatic stellate cells (HSC), the cell type mainly responsible for collagen deposition in the fibrotic liver. Ocoxin was found to reduce the survival of a cell line of immortalized non-tumoral rat HSC in a dose–response fashion and to diminish collagen type I levels. This latter effect was observed even at doses not affecting cell survival, pointing to an antifibrogenic action for the supplement. The decrease in viability exerted by Ocoxin on HSC correlated with an increase in histone-associated fragments in the cytoplasm and with increased activity of caspase-3, indicating the induction of apoptosis. To determine the molecular mechanisms mediating Ocoxin-induced apoptosis, the activation of members of the MAPK family was analyzed. Incubation of HSC with Ocoxin caused a transient and dramatic enhancement on ERK, JNK, and p38 MAPK phosphorylation levels. Using specific inhibitors for these enzymes, p38 MAPK was identified as a key mediator of the apoptotic effect of Ocoxin on HSC.

**Supplementary Information:**

The online version contains supplementary material available at 10.1007/s13105-022-00878-5.

## Introduction

Hepatic fibrosis is a pathological condition that can be triggered by a wide range of agents and is characterized by excessive accumulation of extracellular matrix proteins in the liver. Progressive fibrosis can eventually result in cirrhosis, liver failure, or hepatocellular carcinoma, and it is considered the hallmark of chronic liver injury [1, 5]. Several cell types regulated by a complex net of factors are involved in the development of liver fibrosis. Among them, activated hepatic stellate cells (HSC) are the cell type mainly responsible for extracellular matrix deposition and therefore a key target for antifibrogenic strategies [29]. Oxidative stress due to agents like alcohol or the profibrogenic cytokine transforming growth factor-beta (TGF-β) or caused by infiltrating immune cells has been shown to mediate the development of hepatic fibrosis and to directly contribute to collagen production by HSC [2, 26]. A relevant number of studies have evaluated the effects of different antioxidants, either by themselves of combined with other agents, in the progression of liver fibrosis, showing in most instances moderate but significant beneficial effects [15, 35], and in some cases even detrimental outcomes [21].

Ocoxin is a nutritional supplement which contains agents that have been reported to cause antioxidant and/or hepatoprotective actions, such as glycyrrhizic acid [16, 19], ascorbic acid [8], or epigallocatechin [28]. Clinical studies carried out in patients with chronic hepatitis C [9, 32] and cirrhosis [34] have demonstrated Ocoxin treatment to improve oxidative stress and immunological parameters as well as to diminish the extent of liver fibrosis. In patients with nonalcoholic fatty liver disease (NFLD), the administration of Ocoxin resulted in an improvement in some histological markers of steatosis and inflammation [33]. Additionally, the administration of this nutritional supplement inhibited the proliferation of human hepatocellular carcinoma cell lines and reduced tumor progression in animal models of liver cancer [6]. Moreover, the induction of apoptosis has also been described in metastatic liver lesions derived from an experimental animal model of colorectal carcinoma treated with Ocoxin [17]. To date, the mechanisms through which Ocoxin exerts these effects have not been fully established. Taking into account the key role played by HSC in the development of liver fibrosis, the aim of the present study was to determine the potential effects of Ocoxin on HSC and to analyze the molecular mechanisms involved.

## Materials and methods

### Reagents

Cell culture reagents were obtained from Gibco (Thermo Fisher Scientific, Inc., Waltham, MA, USA). 5–6-Chloromethyl-2′,7′-dichlorohydrofluorescein diacetate (CM-H2DCFDA) was from Molecular Probes (Eugene, OR). SB203580 and PD98059 were purchased from Calbiochem® (Germany). L-JNKI1 was from ALEXIS® Biochemicals (Lausen, Switzerland). General chemical reagents were purchased from Sigma Aldrich (Merck KGaA, Darmstadt, Germany) unless otherwise specified. Cell culture plastics were obtained from Corning (Thermo Fisher Scientific, Inc., Waltham, MA, USA).

Ocoxin was from Catalysis S.L. (Madrid, Spain). As shown in Table 1, Ocoxin is a formulation that includes the components mentioned in the list.

**Table 1:** List of the components included in Ocoxin oral solution (OOS) formulation; the final amount of each component in 100 mL of the solution [23]

Plant extracts	Components quantity
Glycyrrhiza glabra extract	200 mg
Green tea extract (EGC)	25 mg
Cinnamon extract	3 mg
*Vitamins*	
Ascorbic acid (Vit. C)	120 mg
Pyridoxine (Vit. B6)	4 mg
Cyanocobalamin (Vit. B12)	2 μg
Folic acid (Vit. B9)	400 μg
Calcium pantothenate (Vit. B5)	12 mg
*Amino acids*	
Glycine	2000 mg
Arginine	640 mg
Cysteine	204 mg
*Sugars*	
Glucosamine	2000 mg
Sucralose	24 mg
*Other components*	
Malic acid	1200 mg
Zinc sulfate	80 mg
Manganese sulfate	4 mg
Sodium benzoate	100 mg
Potassium sorbate	100 mg
Maracuya aroma	50 mg

### Cell culture and materials

The experiments were performed using the HSC line CFSC-2G. This non-tumoral cell line was obtained after spontaneous immortalization of HSC isolated from a rat CCl4-cirrhotic liver and is characterized by low basal levels of expression of type I collagen genes and by the presence of mRNA for nestin and α-SMA. Therefore, it can be considered as a “transitional” HSC, in which the activation process is already initiated. Cells were cultured in MEM supplemented with 10% fetal bovine serum (FBS) and non-essential amino acids for 36 h after which the medium was replaced with a serum-free medium. Treatments were carried out 12 h later. HSC were treated with Ocoxin for the indicated times and dilutions (in culture media). In some experiments, cells were pretreated for 30 min with either 10 μM SB203580, PD98059, or L-JNKI1. The specificity of these inhibitors at concentrations used in the present study has been demonstrated in previous reports [31]. Protein inhibitors were initially dissolved in dimethyl sulfoxide (DMSO) (“vehicle”; ≥ 99.9%, Merck, Darmstadt, Germany). The same amount of DMSO (vehicle) was added to control cells to discard possible effects related to the vehicle. The LX-2 cell line, an immortalized human hepatic stellate cell line (Merck, Darmstadt, Germany) was cultured in high glucose DMEM supplemented with 2% heat-inactivated FBS and 1% penicillin/streptomycin (5000 U/mL) (Lonza, Basel, Switzerland). The human hepatoma cell lines HuH7 and HepG2 were obtained from the American Type Culture Collection (Rockville, MD) and cultured in DMEM supplemented with 10% FBS, l-glutamine, and antibiotics. Thirty-six hours after cells were plated, the medium was replaced by DMEM supplemented without FBS, and treatments were carried out 12 h later. Freshly isolated hepatocytes were obtained by liver collagenase perfusion from male Wistar rats. Isolated hepatocytes were resuspended and plated in MEM supplemented with 10% FBS, antibiotics, and non-essential amino acids. They were cultured in plates for two hours. After this time, the culture media was replaced by freshly culture media in order to remove dead cells. The hepatocytes were then cultured for 24 h, and afterwards, the culture media was replaced by a medium without FBS, and treatments were carried out 12 h later.

### Neutral red assay

Cell viability was analyzed using neutral red (NR, 3-amino-7-dimethylamino-2-methylphenazin), a weak cationic stain which is actively concentrated by viable cells and accumulates in lysosomes. For NR assay 4 × 10^4^ CFSC-2G, LX2, hepatocytes, HUH7, or HepG2 cells were seeded in a 96-well plate and treated during 24 h with the indicated dilutions of Ocoxin. Then, 50 μl of NR 1 mg/mL:NaCl 1.8% (1:1) was added and cells were incubated for 90 min, at 37 °C in a 5% CO_2_ atmosphere. Cells were washed twice with PBS and the vital dye incorporated by viable cells was released by adding 100 μl of NaH_2_PO_4_ 0.05 M and 50% ethanol. The absorbance of the samples was measured at 540 nm and referred to the absorbance of untreated cells. Values are mean ± SD of at least triplicate data from three independent experiments.

### Determination of oligonucleosomal (histone-associated) DNA fragments

The presence of soluble histone-DNA complexes was measured by the Cell Death Detection Assay (Roche® Life Science Products, Merck KGaA, Darmstadt, Germany). For this assay, HSC were seeded on 96-well plates at a density of 4 × 10^4^ cells/well. Cell death ELISA assays was performed according to the manufacturer’s instructions. Specific enrichment of mono- and oligonucleosomes released into the cytoplasm (enrichment factor, EF) was calculated as the ratio between the absorbance values of the samples obtained from treated and control cells.

### Measurement of caspase-3 activity

The caspase 3 colorimetric assay kit (Sigma Aldrich Merck KGaA, Darmstadt, Germany) was used to detect caspase-3 activity. The assay is based on the spectrophotometric detection of the chromophore *p-nitroaniline* (pNA) which is released after cleavage of the substrate acetyl-Asp-Glu-Val-Asp p-nitroanilide (Ac-DEVD-pNA) by caspase-3. The presence of pNA was measured with an ELISA plate reader (Multiskan Ex, Thermo Electron Corporation) reading the absorbance at 405 nm. Absorbance values of treated samples were compared with those of control samples to determine the increase of caspase-3 activity. For this assay, 6 × 10^5^ cells were seeded in 60-mm plates and treated for 6 h with Ocoxin at different dilutions.

### Measurement of intracellular reactive oxygen species (ROS) levels

ROS were measured using the fluorescent probe CM-H_2_DCFDA. For these experiments, HSC were grown in MEM without phenol red. For time course studies, HSC were plated to sub-confluence in 60-mm culture dishes, treated at different times with Ocoxin and then incubated for 20 min with 5 μM CM-H_2_DCFDA at room temperature. Fluorescence was analyzed in a Cytofluor 2350 (excitation at 485 nm, emission at 530 nm).

### Western blot analysis

HSC were treated as described above and proteins extracted in RIPA buffer (25 mM Tris–HCl, 150 mM NaCl, 0.1% w/v SDS, 1% w/v sodium deoxycholate, 1% v/v IGEPAL). The protein concentration of the resultant samples was determined by BCA (bicinchoninic acid assay, Sigma Aldrich Merck KGaA, Darmstadt, Germany). For immunoblotting analysis, equal amounts of protein were electrophoresed on SDS–polyacrylamide gels and transferred on membranes. Membranes were incubated with a specific antibody to phospho-ERK (Promega), phospho-JNK, phospho-p38 MAPK, type I collagen (Rockland Inmunochemicals) or actin (Santa Cruz Biotechnology, Santa Cruz, CA, USA). After incubation with the secondary antibody conjugated to horseradish peroxidase (Promega), immunoreactive proteins were detected by an enhanced chemiluminescent system (ECL, Amersham International plc, Little Chalfont, UK).

### Statistical analysis

Data were analyzed using one-way ANOVA to determine differences between all independent groups. When significant differences were obtained (*p* < 0.05), differences between groups were tested using Dunnett’s multiple comparisons test. Graphs were generated using GraphPad Prism 9 (GraphPad, San Diego, CA, USA).

## Results

### Ocoxin diminishes cell viability of HSC

**Fig. 1:**
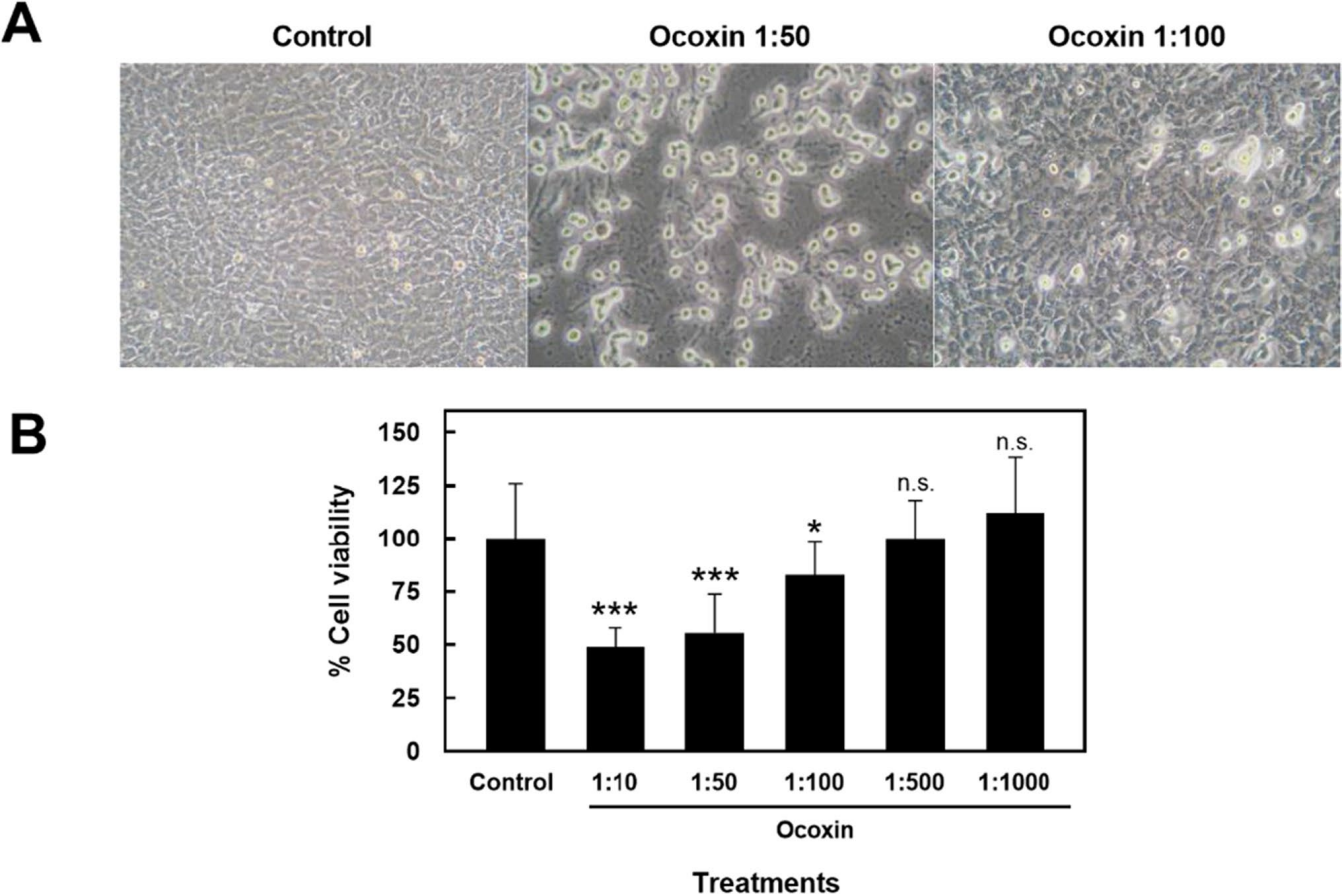
Analysis of HSC viability in response to Ocoxin. HSC were treated for 24 h with the indicated dilutions of Ocoxin. **A** Morphology by light microscopy of control and Ocoxin-treated HSC. Original magnification × 200. **B** Cell viability in response to Ocoxin determined by neutral red assay. Control represents non-treated cells. Each bar represents the mean ± SD of percentage viability fold change compared to control, of triplicate data from four independent experiments (*p < 0.05, ***p < 0.001, vs control)

The first experiments aimed to determine the effect of Ocoxin on cell survival of the HSC line CSFC-2G. Cell cultures were exposed for 24 h to increasing dilutions of Ocoxin, ranging from 1:10 to 1:1000. The morphological effects of Ocoxin on HSC were evaluated by light microscopy for the 1:50 and 1:100 dilutions. As shown in Fig. 1A, Ocoxin caused, in a dose–response fashion, a highly refringent and round-shaped cells appearance. Cell viability assay, using neutral red, showed a correlation of morphological changes and loss of cell viability, which was significant for 1:10–1:100 dilutions (Fig. 1B).

**Fig. 2:**
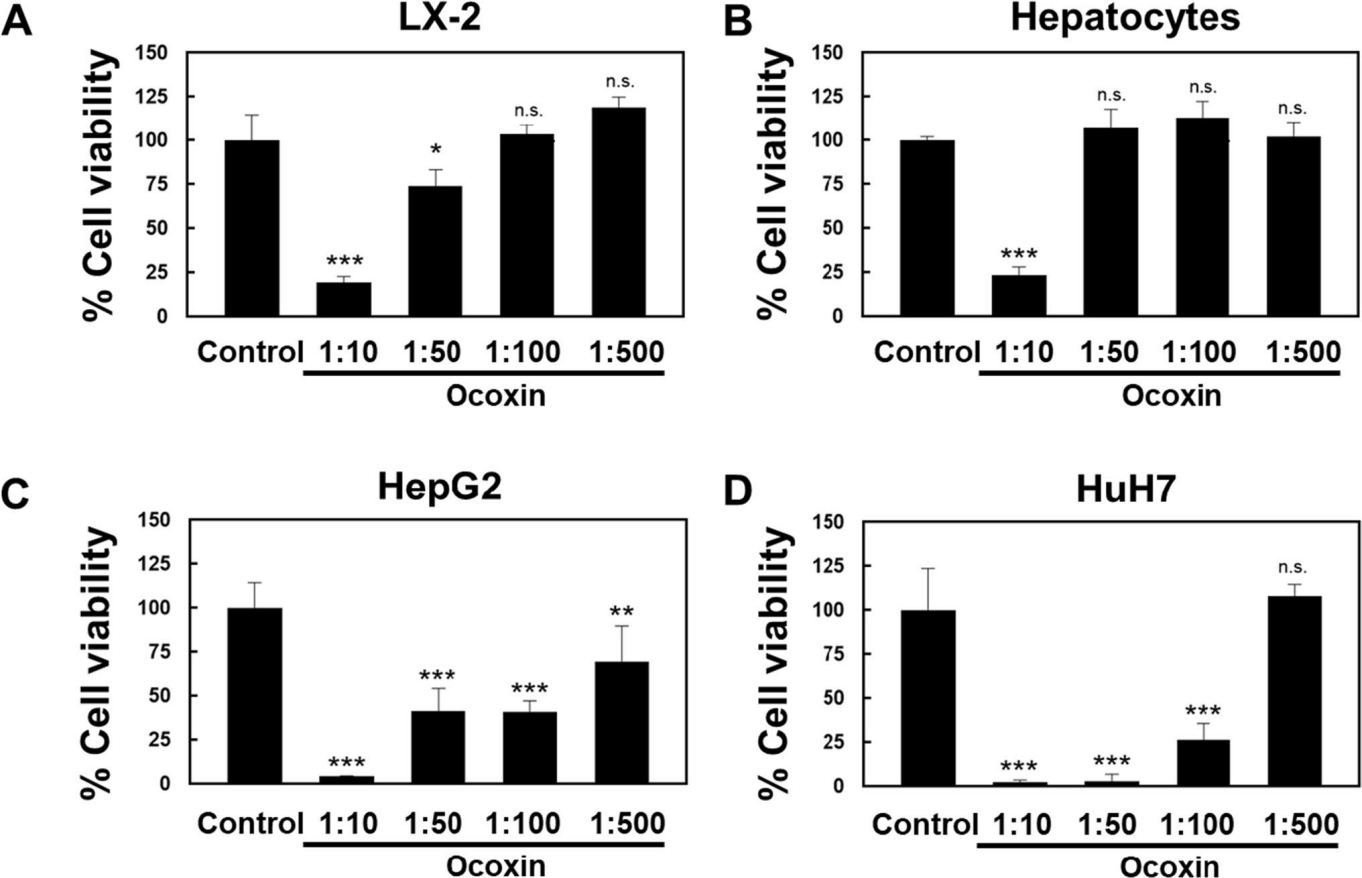
Cell viability in response to Ocoxin treatment for 24 h determined by a neutral red assay in; **A** human hepatic stellate cells LX-2. **B** Normal rat hepatocytes. **C** Human hepatoma cell line HepG2. **D** Human hepatoma cell line HuH7. Control represents non-treated cells. Each bar represents the mean ± SD of percentage viability fold change compared to control of triplicate data from four independent experiments (*p < 0.05, **p < 0.01, ***p < 0.001, vs control)

The reduced viability of HSC was confirmed in the human HSC cell line LX-2 and the hepatoma cell lines HepG2 and HuH7, employing the same dilutions of the nutritional supplement. Noteworthy, only the lowest dilution of Ocoxin significantly affected the cell viability of primary hepatocytes (Fig. 2).

### Ocoxin reduces the production of type I collagen in HSC

**Fig. 3:**
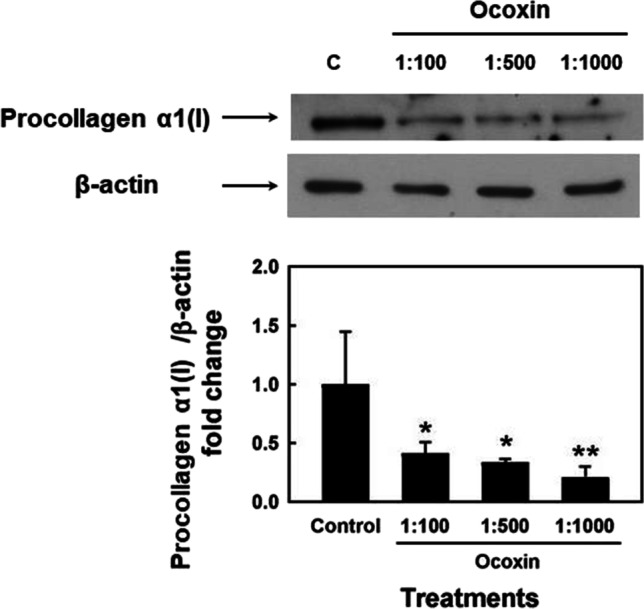
Effect of Ocoxin on procollagen α1(I) levels in HSCs. HSCs were incubated with the indicated dilutions of Ocoxin for 24 h. Protein extracts were analyzed with Western blot using specific antibodies for procollagen α1 (I) and β-actin. β-Actin protein levels were used as the loading control. Results are representative of at least three independent experiments. (*p < 0.05, **p < 0.01, vs control)

The analysis of the effect of Ocoxin on the production of type I collagen in HSCs was carried out by Western blot determination of its precursor form procollagen α1 (I) levels. To this purpose, a 24-h treatment was conducted with different dilutions of Ocoxin, including a dilution with an effect on cell viability (1:100) and dilutions with no effect on cell viability (1:500 and 1:1000). As shown in Fig. 3, collagen type I levels were decreased in all cases, suggesting an antifibrogenic effect even at low doses that did not affect cell viability.

### Ocoxin induces apoptosis in HSC

**Fig. 4:**
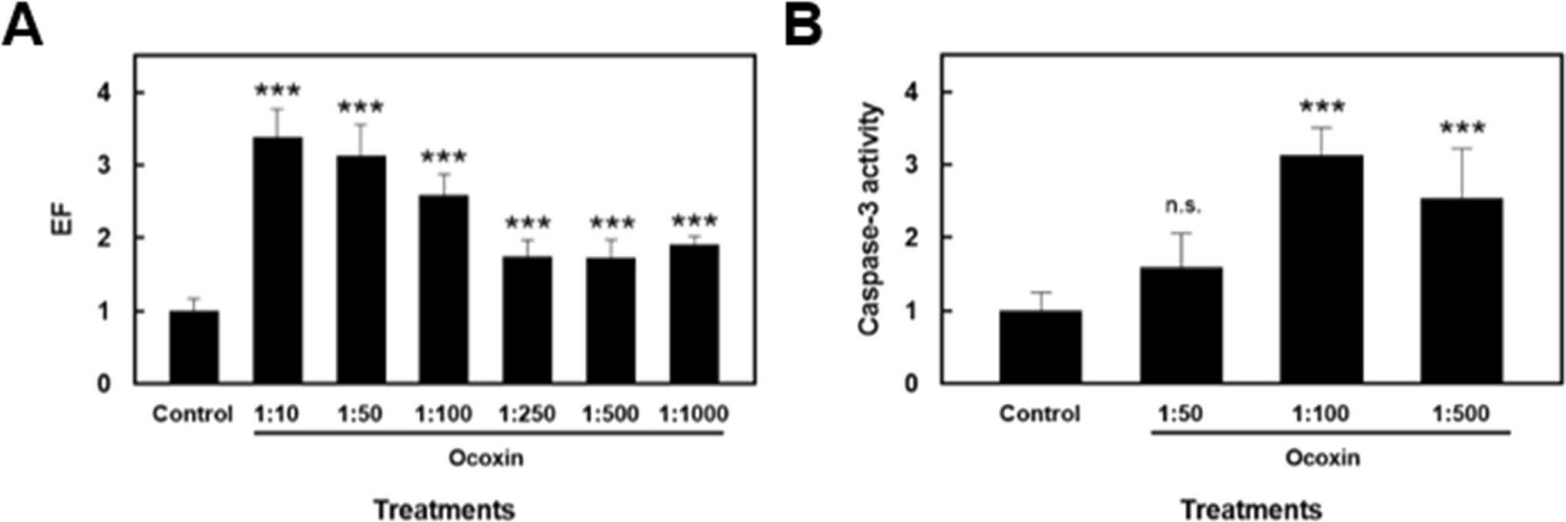
Analysis of HSC apoptosis in response to Ocoxin. **A** Determination of oligonucleosomal fragments in cytoplasmic extracts from HSC treated with Ocoxin. Oligonucleosomal fragments content was determined by ELISA and expressed as enrichment factor (EF), as described in Materials and methods. **B** Caspase-3 activity levels were analyzed in HSC treated with Ocoxin for 6 h, using a commercial colorimetric kit, as indicated in the “Materials and methods” section. Each bar represents the mean + SD of quadruplicate determinations from at least two independent experiments (***p < 0.001, vs control)

The presence of histone-associated oligonucleosomal fragments in the cytoplasm reflects the extent of DNA fragmentation and nuclear disruption that are characteristic of apoptosis. To determine whether the decrease in cell survival caused by Ocoxin was due to the induction of apoptosis, cytoplasmic levels of oligonucleosomal fragments were analyzed by ELISA in HSC treated with several dilutions of Ocoxin. A significant enhancement on oligonucleosomal fragment content was observed in response to treatment with all the dilutions, being higher for 1:10 to 1:100 dilutions (Fig. 4A).

Caspases are a family of cysteine proteases which are essential mediators of the apoptotic process. Caspase-3 is an ultimate effector of caspase-mediated apoptosis. To study whether Ocoxin-induced apoptosis is caspase-mediated, the activity of this enzyme was measured with a commercial kit in HSCs incubated with the supplement for 6 h. As observed in Fig. 4B, treatment of the HSCs with different dilutions of Ocoxin caused an increase of caspase-3 activity, suggesting a caspase-dependent cell death induced by Ocoxin.

In order to determine the possible involvement of reactive oxygen species (ROS) in the molecular mechanisms that could mediate the apoptotic effect of Ocoxin, intracellular levels of ROS were analyzed using the fluorogenic probe CM-H_2_DCFDA in HSC treated for 1–6 h with these different dilutions of this nutritional supplement (Supplementary Figure S1). However, only a slight and transient increase in intracellular levels of ROS was observed 6 h after treatment, pointing to a ROS-independent apoptotic mechanism.

### Role of members of the MAPK family on apoptosis of HSC induced by Ocoxin

**Fig. 5:**
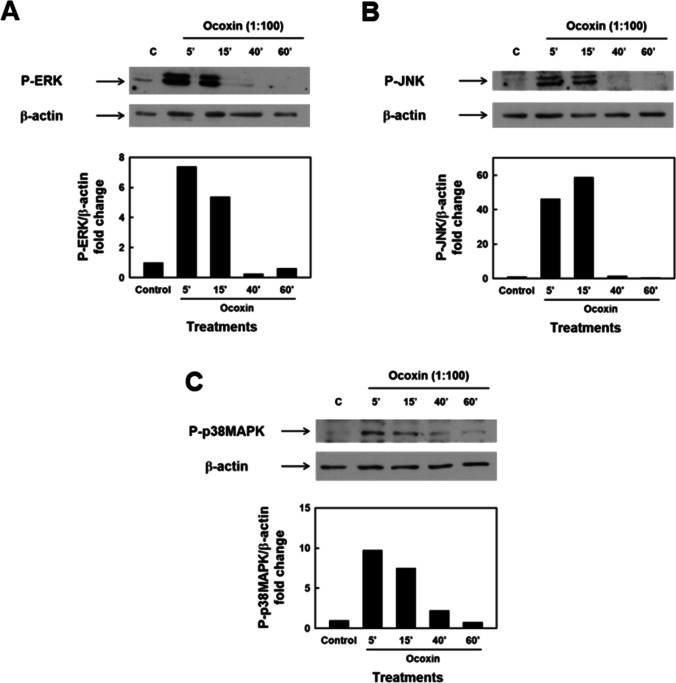
Analysis of ERK, JNK, and p38MAPK signaling pathways in HSC treated with Ocoxin. HSC were treated with Ocoxin 1:100 dilution for 5–60 min and Western blot analysis for the phosphorylated active forms of ERK, JNK, and p38MAPK was carried out using specific antibodies. β-Actin protein levels were used as the loading control. Results are representative of at least three independent experiments

Once established the type of cell death induced by Ocoxin, we next evaluated the involvement of the main MAPK in the apoptotic effect of the nutritional supplement. Time course treatments using a 1:100 dilution of Ocoxin were carried out and the phosphorylated forms of ERK, JNK, and p38MAPK were detected by Western blot analysis using specific antibodies. As shown in Fig. 5, increased phosphorylation of ERK, JNK, and p38MAPK was observed reaching maximum values 5–15 min after treatment.

**Fig. 6:**
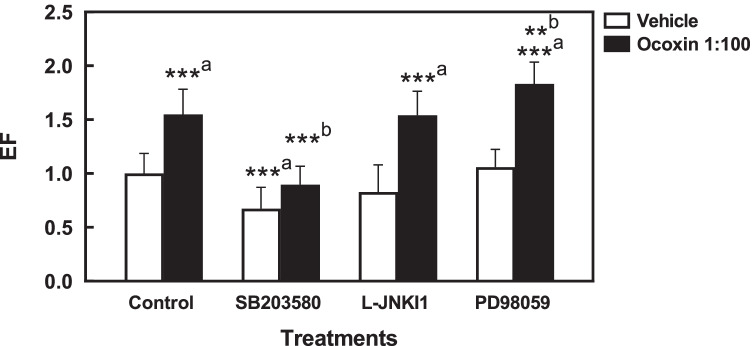
Role of p38 MAPK, JNK, and ERK signaling on HSC apoptosis induced by Ocoxin. HSC were pretreated for 30 min with 10 μM SB203580 (p38MAPK inhibitor), L-JNKI1 (JNK inhibitor) or PD098059 (MEK1 inhibitor), and treated with 1:100 dilution of Ocoxin. Determination of oligonucleosomal fragments in cytoplasmic extracts was carried out by ELISA and expressed as enrichment factor (EF), as described in the “Materials and methods” section. Each bar represents the mean + SD of quadruplicate determinations from at least three independent experiments (**p < 0.01, ***p < 0.001, a, vs control; b, vs Ocoxin-treated cells)

To establish whether activation of the enzymes analyzed was involved in the apoptotic action of Ocoxin, HSC were pretreated for 30 min with pharmacological inhibitors for p38MAPK (SB203580), JNK (L-JNKI1), or ERK (PD098059, inhibitor of MEK1) prior incubation with 1:100 dilution of Ocoxin, and the extent of apoptotic cell death was determined as above. Inhibition of JNK did not alter the cytosolic levels of oligonucleosomal fragments enhanced by Ocoxin, suggesting this enzyme is not involved in the apoptotic response. Pretreatment with the inhibitor for ERK activation, on the other hand, had a potentiating effect on the apoptotic action of Ocoxin, indicating that ERK could have an antiapoptotic or proliferative role on HSC. Finally, the inhibitor for p38 MAPK significantly prevented the apoptotic effect of Ocoxin, demonstrating p38 MAPK is involved in the apoptotic response (Fig. 6).

## Discussion

**Fig. 7:**
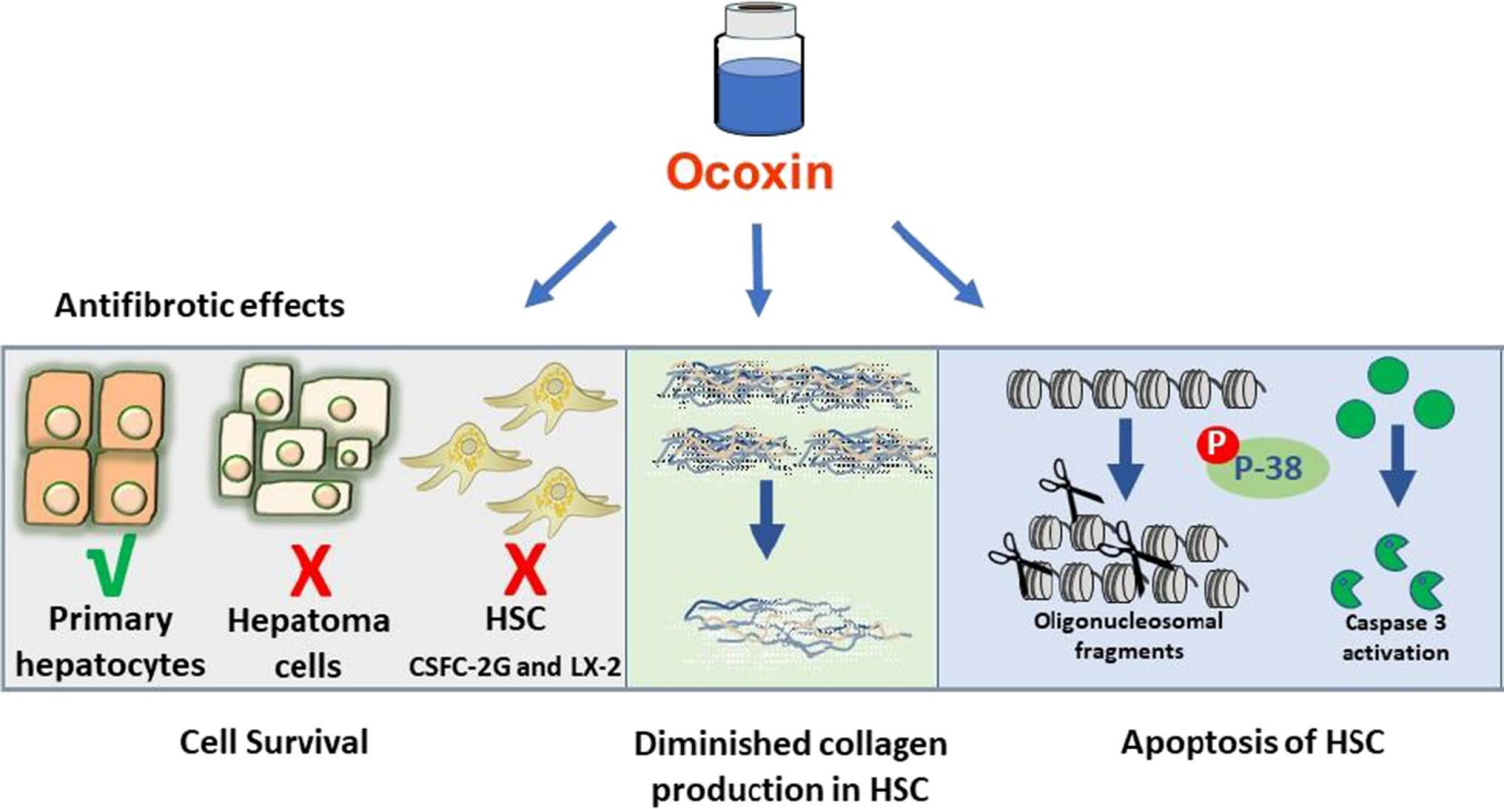
Schematic diagram summarizing the main findings of the study

Liver fibrosis is considered the result of the dysregulation caused by chronic injury of an otherwise transient process, the wound healing response, and therefore a pathological situation that could be eventually reversible. In fact, either fibrotic or cirrhotic regression has been described in most chronic liver diseases, including autoimmune hepatitis, NASH, and viral hepatitis [13]. Together with other processes, such as cellular senescence [14], clearance of activated HSC through apoptosis has been demonstrated to contribute to fibrosis regression both in animal models [11] and clinical studies [20]. These observations confirm previous results demonstrating the contribution of enhanced HSC survival to progressive fibrosis [22, 24]. In the present study, Ocoxin was found to present antifibrotic activity as it induced apoptosis and reduced collagen production in a cell line of immortalized rat HSC (Fig. 7). The cell line used (2G-CFSC) can be considered as a “transitional” HSC, in which the activation process is already initiated [31] and therefore a good model for early fibrosis. A pro-apoptotic effect of Ocoxin on activated HSC could explain some of the beneficial outcomes found in clinical studies carried out with hepatitis C patients, in which treatment with Ocoxin either by itself [9] or combined with interferon alpha-2b and ribavirin caused a reduction of fibrosis [32]. Regarding NAFLD, Ocoxin seemed to have a more relevant effect on inflammation and in histological markers of steatosis rather than in fibrosis [33].

Endogenous production of reactive oxygen species (ROS) is altered in HSC in response to TGF-β, acetaldehyde [10], or leucine [25], leading through different mechanisms to an increased fibrogenic activity. On the other hand, prooxidant agents like menadione or SIN-1 have been described to induce apoptosis of HSC, an effect that was prevented by pretreatment with antioxidants [18]. Although ROS can regulate HSC biology and Ocoxin has been shown to improve oxidative parameters in vivo, the apoptotic effect of this compound on HSC does not seem to be mediated by alterations in ROS intracellular content since only some slight changes at certain doses were observed (Supplementary Figure S1). In fact, there is increasing evidence indicating that many beneficial effects of agents with antioxidant potential are due to mechanisms not directly related to ROS scavenging, such as regulation of transcription factors or signaling pathways [4, 27].

Mitogen-activated protein kinase (MAPK) superfamily members p42/p44 MAPK (ERK), and the stress-activated members c-Jun amino-terminal kinase (JNK), and p38 MAPK, are mediators of the cellular responses to stimuli like growth factors or stress signals. More specifically, JNK and p38 MAPK have been shown to exert pro-apoptotic effects in different cell types. We found Ocoxin to induce early and transient activation of ERK, JNK, and p38MAPK. Although both JNK and p38 MAPK have been previously demonstrated to be involved in apoptosis of HSC in response to different agents [7, 12], only p38 MAPK seemed to mediate the effect of Ocoxin since inhibition of this enzyme but not of JNK significantly prevented apoptosis of HCS. Interestingly, p38 MAPK plays also an important role in the profibrogenic response of HSC, mediating the activation process and both basal and TGF-β-induced production of collagen type I [3, 30, 31]. Our results give additional evidence of p38 MAPK being a key mediator of the biology of HSC, involved not only in their fibrogenic activity but also in the molecular mechanisms leading to their programmed cell death.

### Supplementary Information

ESM 1 (PPTX 103 KB)

## Data Availability

The data that support the findings of this study are available from the corresponding author, Dra María J. Iraburu, upon reasonable request.
