# Supplementary material for: Antifibrogenic and apoptotic effects of Ocoxin in cultured rat hepatic stellate cells
Source: J Physiol Biochem. 2022 Mar 3;79(4):881–90. doi: 10.1007/s13105-022-00878-5 (PMC10635942; doi:10.1007/s13105-022-00878-5)
Supplement: Supplementary file 1 — (PPTX 103 KB) [file 13105_2022_878_MOESM1_ESM.pptx]

## Slide 1
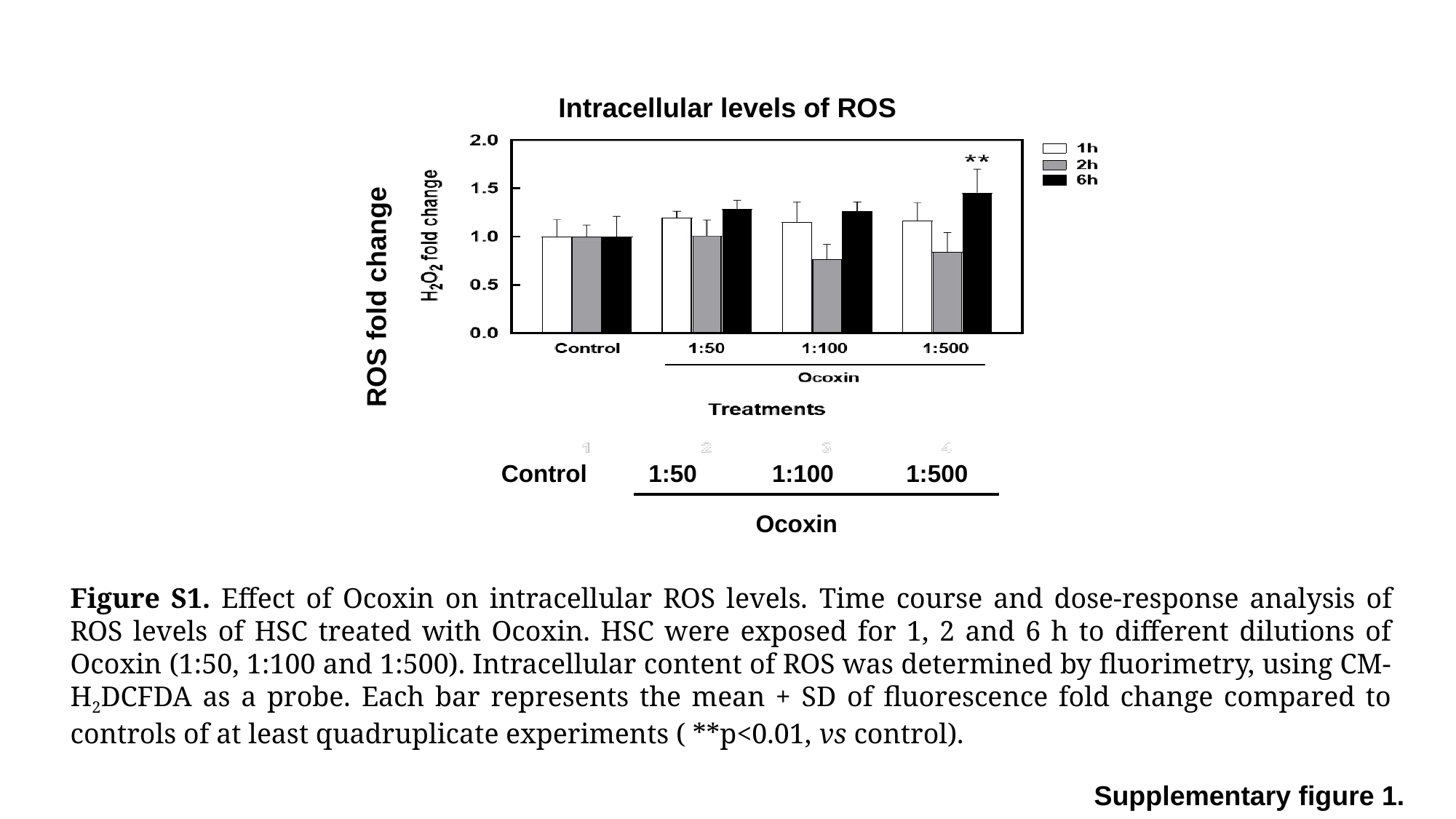

Intracellular levels of ROS
ROS fold change
Control
1:50
1:100
1:500
Ocoxin
Figure S1. Effect of Ocoxin on intracellular ROS levels. Time course and dose-response analysis of ROS levels of HSC treated with Ocoxin. HSC were exposed for 1, 2 and 6 h to different dilutions of Ocoxin (1:50, 1:100 and 1:500). Intracellular content of ROS was determined by fluorimetry, using CM-H2DCFDA as a probe. Each bar represents the mean + SD of fluorescence fold change compared to controls of at least quadruplicate experiments ( **p<0.01, vs control).
Supplementary figure 1.
